# Indigenous people should decide on matters of access to archival information

**DOI:** 10.3402/ijch.v75.32593

**Published:** 2016-06-27

**Authors:** Ry Moran

**Affiliations:** National Centre for Truth and Reconciliation, University of Manitoba, Winnipeg, MB, Canada. Email: ry.moran@umanitoba.ca

Access to information and protection of personal privacy are enshrined as two pillars of a democratic society. However, a critical examination of who has access to information, why they have access to that information and who has the decision-making authority to determine what is released through a lens of Indigenous rights, self-determination and sovereignty reveals that for many years, Indigenous peoples have been excluded from determining who controls access to information about them.

While Canada has amassed close to twenty *kilometers* of paper records detailing the relationship between the state and Indigenous people through the “Indian Affairs” files at Library and Archives Canada, Indigenous peoples have had little to no say regarding the use, disclosure or access to that information.

The National Centre for Truth and Reconciliation (NCTR) is tasked with honouring, sharing and preserving a sacred collection of materials that was amassed through the work of the Truth and Reconciliation Commission (TRC) and the mandatory obligation placed on parties to the Indian Residential Schools Settlement Agreement to share their documents with the TRC.

The resulting archive, now housed at the NCTR, consists of millions of records, many of which contain sensitive personal information on a range of matters related to health, schooling, interactions with police and other matters. The transfer of these records from the control of the church and government archives into an institution governed and led by Indigenous peoples represents a fundamental shift in the balance of power regarding who has access, the reasons for access and concepts of ethical and respectful stewardship.

Some of the materials collected by the TRC – such as the school files from the RG10 series at Library and Archives Canada – are currently in the public domain. Microfilmed in the 1960s, distributed across the country and later digitized and placed online, these records contain personal information about students and their families. These records were made public in an era before present day concepts of privacy and access were developed in this country.

The NCTR too has placed many of these records online in an effort to fulfil our mandate to broadly inform the Canadian public and to make the records collected by the TRC “as accessible as possible.” However, just as the authors of this paper are left with a sense of unease about the ready and free availability of materials that would ordinarily be protected under privacy legislation, so too is the centre wrestling with many of these same issues.

Since receiving the materials from Library and Archives Canada, we have undertaken a thorough review of the materials in the school series. This review revealed that many of the school series records contain information that could cause discomfort, harm or embarrassment to individual survivors. For example, it was commonplace for admissions records to track whether students had syphilis. Other records often feature highly racialized comments about students and their families written by Indian agents, teachers or principals.

We now believe there are compelling reasons to take a far more cautious approach to the dissemination of archival materials than has been conducted in the past. As a result, we have removed some of these documents from the public-facing website of the centre.

That said, the balance between access and privacy is a complicated matter. Canada as a nation is woefully ignorant of the realities of student life in the residential schools. The entire reason documents were collected by the TRC was to ensure Canadians could grow and learn from a full and complete history of the residential school – ugliness and all.

Papers, such as this one, present new information that reveals the fundamental misalignment between government policies and the health and well-being of Indigenous people. Such papers are critical in advancing our understanding of the harms and effects the schools had on young Indigenous children.

Through a recent series of community engagement sessions held by the NCTR, survivors and intergenerational survivors reiterated these messages in meetings across the country: ensure that Canadians know the truth and ensure that Canadians know what survivors had to endure as young children.

Striking a balance between access and privacy is exceptionally tricky in this regard, and, through work with survivors and the governing circle of the NCTR, we too are finding our own path forward through this. What can be said for certain however is that re-victimizing survivors through the inappropriate use or disclosure of information is not an option. Breaching whatever fragile trust has been created through these processes of reconciliation will erode confidence in research and record keeping.

But those risks do not mean ongoing research is not critical. On the contrary, the need for research, conducted in respectful ways sensitive to the needs of survivors, has never been greater.

Being guided by the principle of reconciliation articulated by the TRC – the establishment and maintenance of mutually respectful relationships – means that Indigenous peoples can and must have much greater say in who has access to what records. This may also mean that some materials currently in the public domain should simply no longer be so.

Indigenous peoples need to lead this conversation. In the meantime, we need to continue to use materials carefully and cautiously, engaging with the communities affected, consulting relevant stakeholders and challenging the fundamental power structures that lay beneath the questions of who has access to and control over records by and about Indigenous people. This approach is a path to reconciliation itself.

*Ry Moran* Director National Centre for Truth and Reconciliation University of Manitoba Winnipeg, MB, Canada Email: ry.moran@umanitoba.ca

